# The effectiveness of hyperbaric oxygen in patients with idiopathic sudden sensorineural hearing loss: a systematic review

**DOI:** 10.1007/s00405-018-5162-6

**Published:** 2018-10-15

**Authors:** Burak Eryigit, Fuat Ziylan, Furkan Yaz, Hans G. X. M. Thomeer

**Affiliations:** 1 Department of Otorhinolaryngology and Head and Neck Surgery, University Medical Center Utrecht, Heidelberglaan 100, 3584 CX Utrecht, The Netherlands; 2 Department of Otolaryngology-Head and Neck Surgery, Ear & Hearing, Amsterdam UMC, Vrije Universiteit Amsterdam, De Boelelaan 1117, Amsterdam, The Netherlands; 3 Brain Center Rudolf Magnus, Utrecht, The Netherlands

**Keywords:** Hyperbaric oxygen, Sensorineural hearing loss, Corticosteroids

## Abstract

**Objective:**

To evaluate the effectiveness of hyperbaric oxygen in the treatment of patients with idiopathic sudden sensorineural hearing loss (ISSHL).

**Data sources:**

An Embase, MEDLINE and Cochrane search were utilised to identify various clinical trials on the treatment of ISSHL. Studies that were published between 2002 and 2018 and written in the English, Dutch or German language were included. Search terms included synonyms for idiopathic sudden hearing loss.

**Data synthesis:**

A total of 16 articles were identified regarding hyperbaric oxygen therapy. All patients were evaluated with pure-tone audiometry. A major part of the cases presented with unilateral hearing loss(bilateral hearing loss less than 5%). In several studies, the average of the mean hearing gain at five contiguous frequencies was significantly higher in the hyperbaric oxygen (HBO) therapy and systemic steroid (SS) group in patients with severe or profound hearing impairment. They recorded a significant treatment effect (*p* = 0.005) of HBO + SS therapy on patients with an initial hearing loss of ≥ 81 dB.

**Conclusions:**

On the whole group of ISSHL patients, no significant difference was demonstrated between the intervention and control group. However, in severe or profound hearing-impaired ISSHL patients, significant benefit was observed in the intervention group. These results likely indicate that adding HBO to steroid therapies might be of benefit in cases of severe and profound hearing impairment.

## Introduction

Idiopathic sudden sensorineural hearing loss (ISSHL) may be outlined as hearing impairment over at least 30 dB on three adjacent frequencies. It develops within an interval of maximum 3 days [1]. The incidence ranges from 5 to 20 per 100,000 cases per year [2]. As it is an idiopathic disease, the etiological factors causing this disease are still uncertain. Although viral diseases and vascular deficiencies of the cochlea might be causative for ISSHL, definitive evidence is absent [3].

The prognosis of ISSHL is difficult to determine, if we take into consideration the possibility of spontaneous recovery. This phenomenon seems to occur in 29–78% of patients affected by ISSHL. The shape of the audiogram at onset of the disease may be a predictor of hearing recovery [4].

In clinical practice, systemic steroid therapy (SS) has been the mainstay for ISSHL. More recently, intratympanic steroid (ITS) treatment has emerged and became more popular with otolaryngologists. This is an interesting option due to absence of unfavourable side effects [endocrine problems (i.e., diabetes dysregulation), osteoporosis or weight gain] which are known in the systemic steroid treatment [5].

Hyperbaric oxygen therapy has been commonly used for a long period of time. It was first used in the late 1970s by Goto and Vincey in the treatment of ISSHL [6, 7]. Goto stated that this form of treatment would increase perilymph oxygenation, which is one of the main reasons for utilising this form of treatment with ISSHL patients. Similar results were reported by Lamm et al., who showed that HBO therapy would increase the oxygen tension of the perilymphatic fluids and improve the microcirculation of the inner ear [8].

In theory, a combination of treatments might provide a better outcome for patients with ISSHL. Therefore, this systematic review will investigate evidence for a possible effect of HBO treatment on hearing recovery in ISSHL patients.

## Materials and methods

No ethical committee approval was required for this literature review. The systematic review was conducted according to the preferred reporting items for systematic reviews and meta-analyses (PRISMA) statement [9, 10].

### Search strategy

An electronic search in the Cochrane library, PubMed and EMBASE databases was performed on 19-05-2018. These searches were conducted to identify clinical trials on the treatment of idiopathic sudden sensorineural hearing loss. The PICOS structure was utilised to define the research strategy. It was mandatory for the published studies to be written in the English, Dutch or German language and published between 2002 and 2018. The following search terms were applied: sudden sensorineural hearing loss, sudden deafness and idiopathic sudden hearing loss. Restrictive search terms were hyperbaric therapy, oxygen therapy, corticosteroids and dexamethasone. The detailed search strings can be found in Appendix 1. Titles and abstracts were independently screened by two of the authors (B.E. and F.Y.). Then, the full text of the eligible articles was read. Cross-reference check of included articles was conducted to achieve additional relevant articles. The PRISMA flowchart was used [10].

### Criteria for inclusion

Each study had to be a clinical trial that compared the treatment of sudden sensorineural hearing loss with a control group. Intravenous, oral and intratympanic corticosteroid treatment comparing combinations with or without hyperbaric oxygen therapy, were included. Patients with a sensorineural hearing loss equivalent to at least 30 dB met the inclusion criteria.

### Criteria for exclusion

Several criteria for exclusion were applied: (1) patients who had another cause for their sudden sensorineural hearing loss such as noise, infections or ototoxic medication, (2) conductive hearing impairment, (3) case reports, and comments on articles and reviews, (4) Other languages than English, Dutch or German, (5) patients receiving therapy after 30 days of onset of ISSHL.

### Quality assessment

After screening of title, abstract and full text, the remaining articles were critically appraised for risk of bias. Quality assessment of eligible studies was assessed according to the risk of bias assessment tool for non-randomized studies (RoBANS) [11].

### Data extraction

Study characteristics and outcome data of included studies were extracted, namely: publication year, study design, participants, type of treatment (oral, intravenous or intratympanic steroids), duration and dosage of treatment, follow-up period and pure-tone average (PTA). The primary outcome was the PTA. The committee of the AAO-NHS, established various guidelines for diagnostic criteria in 1995 [12]. To comply with those guidelines, we maintained subsequent hearing levels at four different frequencies (500, 1000, 2000 and 4000 Hz). These frequencies were measured and calculated for every patient individually. Siegel’s criteria were utilised to obtain three different categories of improvement. Category I for cases with complete hearing improvement (final hearing better than 25 dB), category II indicates moderate hearing improvement (> 15 dB gain, final hearing of 25–45 dB) and Siegel category III–IV was defined as no hearing improvement (< 15 dB gain, final hearing poorer than 75 dB) (Table 1) [13].

**Table 1:** Objective outcomes

Intervention	Study	PTA (dB) (SD/IRQ) (PTA at baseline) (Δ%)	PTA level (kHz)	Complete hearing improvement (%)*	Moderate hearing improvement (%)*	No hearing improvement (%)*	Mean hearing gain < 60 dB	Mean hearing gain > 61 dB
Corticosteroid only	Cekin	50.63 (95.85) − 48%	●	11/20 (55%)	4/20 (20%)	5/20 (25%)	●	●
	Topuz	53.1 (70.5) − 22.8%	0.5, 1, 2 and 4	●	●	●	22.33 ± 9.31	16.18 ± 9.00
	Fujimura	56.0 ± 2.58 (62.8 ± 2.2) − 10.8%	0.5, 1, 2 and 4	16/63 (25.4%)	25/63 (39.7%)	22/63 (34.9%)	◘	◘
	Pezzoli	56.0 ± 11.4 (61.0 ± 20.8) − 8.2%	0.5, 1, 2, 4, 6 and 8	0/21 (0%)	3/21 (14.3%)	18/21 (85.7%)	◘	◘
	Alimoglu	50.34 (72.12 ± 20.68) − 30.2%	0.5, 1, 2, 4 and 8	11/58 (19.0%)	13/58 (22.4%)	32/58 (55.2%)	●	●
	Yang	75.77 ± 21.66 (94.64 ± 15.14) − 19.9%	0.5, 1, 2 and 4	17/35 (48.6%)	◘	18/35 (51.4%)	●	●
	Khater	28.1 ± 8.7 (71.94 ± 2.1) − 60.9%	0.5, 1, 2 and 4	6/11 (45.5%)	3/11 (27.3%)	2/11 (18.2%)	●	●
	Liu	● 23.9 ± 2.6	0.5, 1, 2, 4 and 8	93/277 (33.6%)	88/277 (31.8%)	96/277 (34.7%)	17.3 ± 1.4	23.9 ± 2.6
	Tasdoven	◘ (80.7)	0.5, 1, 2 and 4	14/63 (22.2%)	10 /63 (15.9%)	39/63 (61.9%)	◘	◘
	Ajduk	◘ (75.52 ± 9.42)	0.5, 1, 2, 4 and 8	●	●	●	◘	◘
	Callioglu	◘ ◘	0.5, 1, 2, 4 and 8	●	●	●	◘	◘
	Edizer	◘ ◘	0.5, 1, 2, 4 and 8	22/48 (45.7%)	10/48 (20.8%)	16/48 (33.3%)	●	●
	Satar	52.5 ± 28.4 (82.2 ± 18.7) − 36.13%	0.5, 1 and 2	●	●	●	●	●
	Aslan	50 ± 19.6 (70 ± 17.8) − 28.57%	0.5, 1, 2 and 4	●	●	●	●	●
	Narozny	50.62 ± 2.16 (65.4) − 22.60%	0.5, 1 and 2	●	●	●	●	●
	Chi	◘ ◘	0.5, 1, 2, 4 and 8	3/30 (10%)	11/30 (36.7%)	16/30 (53.3%)	●	●
+Hyperbaric O_2_	Cekin	41.75 (81.47) − 48.8%	●	21/36 (58.3%)	8/36 (22.2%)	7/36 (19.4%)	●	●
	Topuz*	37.1 (70.4) − 48.6%	0.5, 1, 2 and 4	●	●	●	22.53 ± 12.68	35.45 ± 22.09
	Fujimura*	64.4 ± 2.7 (74.7 ± 2.3) − 13.8%	0.5, 1, 2 and 4	12/67 (17.9%)	40/67 (59.7%)	15/67 (22.4%)	◘	◘
	Pezzoli*	56.7 ± 15.3 (72.3 ± 27.6) − 21.6%	0.5, 1, 2, 4, 6 and 8	1/23 (4.3%)	5/23 (21.7%)	17/23 (73.9%)	◘	◘
	Alimoglu*	36.85 (63.68 ± 22.97) -42.1%	0.5, 1, 2, 4 and 8	26/61 (42.6%)	14/61 (22.9%)	21/61 (34.4%)	●	●
	Yang	74.76 ± 18.7 (97.26 ± 15.1) -23.1%	0.5, 1, 2 and 4	13/19 (68.4%)	◘	6/19 (31.6%)	●	●
	Khater*	18.1 ± 2.2 (72.86 ± 1.43) -75.15%	0.5, 1, 2 and 4	8/11 (72.7%)	2/11 (18.2%)	1/11 (9.1%)	●	●
	Liu	● (22.7 ± 3.9)	0.5, 1, 2, 4 and 8	17/112 (15.2%)	56/112 (50%)	39/112 (34.8%)	9.9 ± 2.2	22.7 ± 3.9
	Tasdoven	◘ (92.02)	0.5, 1, 2 and 4	3/26 (11.5%)	4/26 (15.4%)	19/26 (73.1%)	◘	◘
	Ajduk	◘ (80.57 ± 5.14)	0.5, 1, 2, 4 and 8	●	●	●	◘	◘
	Callioglu	◘ ◘	0.5, 1, 2, 4 and 8	●	●	●	◘	◘
	Edizer	◘ ◘	0.5, 1, 2, 4 and 8	13/53 (24.4%)	16/53 (30.1%)	24/53 (45.2%)	●	●
	Satar	48.5 ± 32.1 (68.1 ± 24.4) − 28.78%	0.5, 1 and 2	●	●	●	●	●
	Aslan*	30.1 ± 24.0 (68 ± 19.3) − 55.74%	0.5, 1, 2 and 4	●	●	●	●	●
	Narozny*	30.72 ± 2.70 (58.2) − 47.22%	0.5, 1 and 2	●	●	●	●	●
	Chi*	◘ ◘	0.5, 1, 2, 4 and 8	8/30 (26.7%)	16/30 (53.3%)	6/30 (20%)	●	●

● not conducted, ◘ not extractable, *PTA* pure-tone average (kHz), *no*. participants/total participants (%), *IRQ* interquartile range

*Statistically significant difference (*p* < 0.05)

All groups are assessed based upon their hearing level. This hearing impairment has been categorised according to a standard which has been identified by the World Health Organisation (WHO) [14]. No hearing loss has been defined as a hearing level of 26 dB or lower. 26–60 dB hearing level has been classified as mild/moderate impairment. Severe impairment is classified as a hearing loss of 61–80 dB, finally a hearing loss of 81 dB and beyond is graded as profound hearing impairment [14]. The average of the mean hearing gains were calculated and compared within the groups.

### Ethical considerations

Informed consent was obtained from all patients. The participants were given oral information regarding the procedures by their ENT doctors.

## Results

### Search results and selection process

**Fig. 1:**
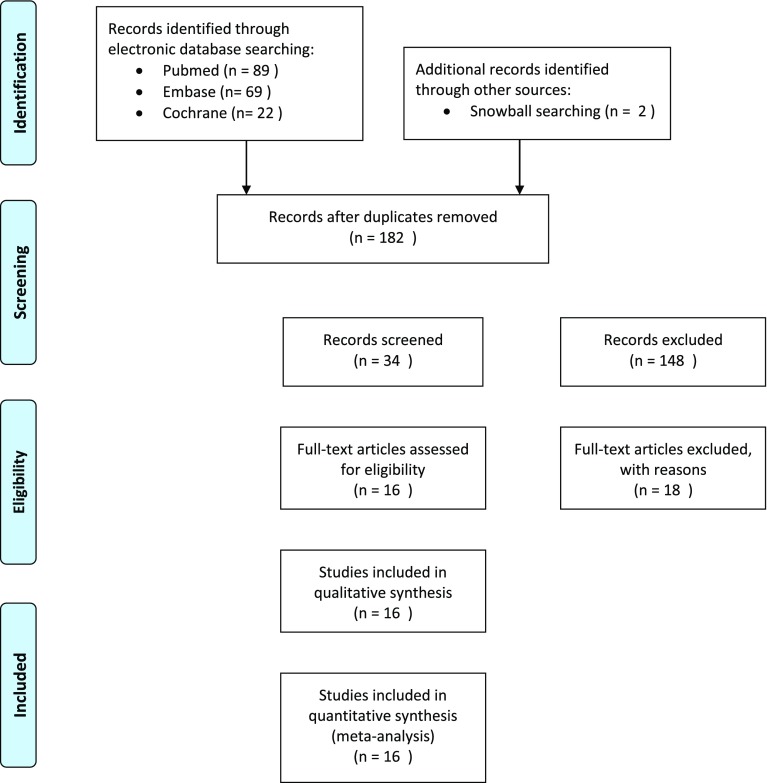
PRISMA flowchart

Figure 1 summarizes the study selection process in a flowchart. We retrieved a total of 182 articles. After title and abstract screening, 34 articles were assessed in full text. In total, 16 articles were included in this systematic review [15–30].

### Quality assessment

According to the RoBANS, the risk of bias of the articles was independently appraised by two reviewers. All eligible articles had satisfactory quality therefore were included for data extraction. Table 2 shows the results of the quality assessment [15–30].

**Table 2:** Critical appraisal

Study	Publication year	Study design	Participants	Selection of participants	Confounding variables	Intervention measurement	Blinding of outcome assessment	Incomplete outcome data	Selective outcome reporting
Cekin	2009	RCT	57	L	H	U	U	H	L
Topuz	2003	RCET	51	L	L	L	U	L	L
Fujimura	2007	RCS	130	H	U	L	H	L	L
Pezzoli	2015	PCS	44	H	L	U	L	L	L
Alimoglu	2011	RCR	219	H	L	L	U	H	H
Yang	2013	RCS	103	H	L	H	H	L	L
Khater	2016	PCS	22	H	L	U	U	L	L
Tasdöven	2016	RCS	89	H	U	L	U	H	L
Ajduk	2017	RCS	93	H	H	U	L	L	L
Callioglu	2015	RCS	44	H	H	L	U	H	L
Liu	2011	RCS	465	H	L	U	H	L	L
Edizer	2015	RCS	205	H	H	L	L	H	L
Satar	2006	RCS	54	H	U	H	L	L	H
Aslan	2002	RCS	50	H	H	L	U	L	L
Narozny	2004	RCS	133	H	L	H	H	L	L
Chi	2017	RCT	60	L	L	L	U	L	L

*Study* first author of the published study, *Publication year* year of publication, *Study design RCS* Retrospective Cohort Study, *PCS* Prospective Cohort Study, *RCET* Randomized Comparison Effectiveness Trial, *RCT* Randomized Clinical Trial, *RCR* Retrospective Case Review, *Participants* number of included relevant participants, *Selection of participants* selection bias caused by inadequate selection of participants, *Confounding variables* selection bias caused by inadequate confirmation and consideration of confounding variable, *Intervention measurement* performance bias caused by inadequate measurement of intervention, *Blinding of outcome assessment* detection bias caused by inadequate blinding of outcome assessment, *Incomplete outcome data* attrition bias caused by inadequate handling of incomplete outcome data, *Selective outcome reporting* reporting bias caused by selective outcome reporting, *L* low, *H* high, *U* unclear

### Baseline characteristics

The total number of operated ears was 1759 of whom 580 were treated with HBO (Table 2). Three different methods of administration were applied. The most performed procedure was the systemic administration for 1278 ears. An intratympanic route was administered for 356 ears. Intravenous was established for 125 ears. Follow-up time varied between 3 weeks and 72 months. 2 out of 16 studies treated their patients with HBO therapy after failure of conventional treatment in ISSHL.

### Systemic steroids with hyperbaric oxygen therapy

Ten suitable trials involving 1295 patients were retrieved (Table 3) [17–25]. A study conducted by Topuz et al., demonstrated an average of the hearing gain at five contiguous frequencies significantly higher in the HBO + SS group for patients with severe or profound hearing impairment (respectively, *p* = 0.014 and *p* = 0.005) [15]. The same result was obtained in the trial guided by Fujimura et al. [16]. Patients suffering from profound hearing loss demonstrated a significantly higher improvement rate in the HBO group than the steroid group (*p* < 0.05).

**Table 3:** Baseline table

Study	Treatment form	Relevant participants	Gender (male)	Mean age (SD or range)	Uni-/bilateral	Research duration (years)	Dosage (mg/kg)	Therapy period (days)	Treatment after onset (days)	Follow-up (months)	Salvage or primary treatment
Cekin et al.	Hyperbaric oxygen therapy + oral prednisolone	36	24	46.8 ± 14.5	Uni (34×)/Bi (2×)	12	1	21	●	●	Primary
	Oral prednisolone	21	8	44.5 ± 16.3	Uni	12	1	21	●	●	Primary
Topuz et al.	Oral prednisolone + HBO	30	14	42.1 ± 13.4	Uni	4	5 + 2.5 ATA	15	< 14	1	Primary
	Oral prednisolone	21	12	40.4 ± 11.2	Uni	4	5	14	< 14	1	Primary
Fujimura et al	Dexamethasone oral + HBO	63	●	53.0 ± 2.1	Uni	3	10 − 5	14	< 30	1	Primary
	Dexamethasone oral	67	●	52.2 ± 1.7	Uni	11	10 − 5	14	< 30	1	Primary
Pezzoli et al.	Dexamethasone IV + HBO	23	16	47.3 ± 13.7	●	2	25 + 2.5 ATA	7 + 15	< 30	0.3	Salvage
	Dexamethasone IV	21	11	54.5 ± 13.1	●	2	25	7	< 30	0.3	Salvage
Alimoglu et al.^a^	Oral prednisolone + HBO	61	●	●	Uni	6	1 mg/kg	21	< 30	2	Primary
	Oral prednisolone	58	●	●	Uni	6	1 mg/kg	21	< 30	2	Primary
Yang chao et al.^a^	Dexamethasone ITS + HBO	19	6	44.68 ± 16.04	Uni	●	5 mg/ml + 2.5 ATA	14	< 14	3	Salvage
	Dexamethasone ITS	35	19	49.71 ± 15.43	Uni	●	5 mg/ml	14	< 14	3	Salvage
Khater et al.	Intratympanic steroid injection + HBO	11	5	45.9 ± 6.9	Uni	1	40 mg/ml + 2 ATA	7 + 20	●	1	Primary
	Intratympanic steroid injection	11	7	45.8 ± 7.14	Uni	1	40 mg/ml	7	●	1	Primary
Liu et al.^a^	Oral prednisolone + HBO	112	20	42.4 ± 14.3	Uni	10	20 + 2.5 ATA	8 + 20	< 14	●	Primary
	Oral prednisolone	277	88	44.0 ± 17.0	Uni	10	20	8	< 14	●	Primary
Tasdoven et al.^a^	Oral prednisolone + HBO	26	◘	50 ± 13	Uni	2	1 (mg/kg)	5	●	3	Primary
	Oral prednisolone	63	◘	50 ± 13	Uni	2	1 (mg/kg)	5	●	3	Primary
Ajduk et al.	Intravenous methylprednisolone + HBO	43	21	53.2 ± 19.4	●	6	240/ 80	8	< 20	1	Salvage
	Intravenous methylprednisolone	50	25	55.5 ± 22.2	●	6	240/ 80	8	< 20	1	Salvage
Callioglu et al.	Oral prednisolone + HBO	21	12	43.1 ± 14.8	Uni	2	60/20 + 2.5 ATA	10/3 + 16	< 30	◘	Primary
	Oral prednisolone	23	18	49.4 ± 12.6	Uni	2	60/20	10/3	< 30	◘	Primary
Edizer et al.	Oral prednisolone + HBO	21	12	45.9 ± 15.4	Uni	5	60 + 2.5 ATA	7 + 20	< 30	6	Primary
	Oral prednisolone	23	18	45.9 ± 15.4	Uni	5	60 + 2.5 ATA	7	< 30	6	Primary
Satar et al.	Oral dexamethasone + HBO	37	25	45.2 ± 15.4	Uni (34×)/bi (3×)	7	4 + 2.5 ATA	15	< 5	●	Primary
	Oral dexamethasone	17	11	46 ± 17.9	Uni	7	4	15	< 15	●	Primary
Aslan et al.	Oral prednisolone + HBO	25	17	48 ± 18.4	Uni	4	1 mg/kg + 2.5 ATA	14 + 13	< 14	2	Primary
	Oral prednisolone	25	15	46.6 ± 17	Uni	4	1 mg/kg	14	< 14	2	Primary
Narozny et al.	Oral prednisolone + HBO	52	31	39 ± 16	Uni	4	60 + 2.5 ATA	14 + 16	◘	●	Primary
	Oral prednisolone	81	32	42 ± 19	Uni	17	30 + 2.5 ATA	14	◘	●	Primary
Chi et al.	Oral prednisolone + HBO	30	27	31.1 ± 12.6	Uni	9	20 + 2.5 ATA	14 + 5	< 10	6	Primary
	Oral prednisolone	30	26	29.5 ± 14.7	Uni	9	20	14	< 10	6	Primary

● not reported, ■ not conducted, ◘ not extractable, *Uni* unilateral, *Bi* bilateral, *Total dosage* total medication given

^a^More than two groups were screened

Tasdoven et al. conducted a trial which indicated that there was no significant difference in the response to treatment between the intervention and control group (*p* < 0.355) [17]. However, they found a statistically significant difference in patients who suffered from profound hearing loss. (*p* = 0.012) A different trial, had the same conclusion with regard to the significance of HBO therapy on patients with severe/profound hearing loss (Table 3) [18].

Three studies, conluded that the addition of HBO did not have a significant impact on the prognosis [19–21]. Satar et al. found a hearing gain in favour of the HBO group. However, this difference was not significant (*p* = 0.754) [22]. In three studies, a significant improvement was found after the addition of HBO therapy [23–25].

### Intravenous steroids with hyperbaric oxygen therapy

Pezzoli et al., assessed 44 patients with ISSHL in their clinical trial [26]. Mean PTA gain was significantly better in the intervention group compared to controls (*p* = 0.01). Recovery for patients suffering from profound and severe hearing loss was significantly associated with a therapy form based upon HBO + IVS (*p* = 0.046).

In Pezzoli’s study, having experienced a moderate form of hearing impairment and increasing days after onset were negatively correlated with recovery. In Alimoglu et al., subjects whom received combination therapy in the first 15 days after onset, showed a statistically significant difference between mean gains (*p* < 0.05) [27].

Ajduk et al. proposed that patients with an initial hearing loss of ≤ 60 dB had significant improvement at 500 Hz [28] Whereas subjects with severe/profound hearing impairment had significant improvements at all six frequencies after they were treated with a combination of IVS + HBO.

### Intratympanic steroid injections with hyperbaric oxygen therapy

Khater et al., treated 22 patients who suffered from ISSHL [29]. The patients were randomly assigned into two groups. The HBO treated group performed significantly better than the ITS group (*p* = 0.0014).

A clinical trial conducted by Chao et al. compared the usage of ITS in combination with HBO therapy [30]. The hearing gain in the ITS + HBO group (22.5 ± 18.7 dB) was greater than in the ITS group (18.87 ± 21.66 dB); however, there was no statistical significant difference between both groups (*p* > 0.05).

## Discussion

In this systematic review, we executed a search of the literature to identify evidence evaluating the effectiveness of hyperbaric oxygen therapy in ISSHL. Our search identified a total of 16 articles [15–30]. In all these articles, treatment groups were separated according to HBO therapy. This additive treatment was applied next to the conventional intervention, namely steroid medication (either intravenous, oral, intratympanic or a combination of these).

### Disease onset and timing of treatment

Various outcomes regarding the effectiveness of HBO are reported: eight out of sixteen articles, concluded that there was a significant difference between the intervention and control group [15, 16, 23–27, 29]. The rationale behind hyperbaric oxygen treatment lies in its vasodilatative effect on the organ of Corti and other inner ear structures (such as the stria vascularis) thereby countering the vascular compromise and oxidative stress which are hypothesized to be major factors playing a role in sustaining ISSHL [31]. This might therefore result in a cumulative treatment effect next to the reduction of inflammation by steroid application. The next relevant clinical question is raised regarding the duration of this destructive inflammatory, vasoconstrictive period directly after disease onset by a yet unknown factor (viral, auto-immune): is there a maximum interval after disease onset within which treatment is useful and effective? International guidelines agree on start of steroid treatment (within 72 h) [32]. In this review, no concordance in start of HBO therapy after disease onset could be found. Most of the effects (if any) are to be expected as soon as possible after symptoms arise. The effectiveness of HBO is time-dependent and effectivity decreases with increasing delay in administration [33]. According to Edizer et al., the rate of no recovery was significantly higher in patients who started treatment after 10 days of onset (*p* = 0.010). The same negative correlation has been recognised by various other reports [34–38].

Though early application of HBOT appears to be a logical advice, practical burden arises. Are there HBO therapy centers available in the region and are these capable of accepting this ‘new patient flow’ for a new indication (hearing recovery after ISSHL). And if so, is it cost effective? There is a considerable percentage of patients known to recover spontaneously [4]. This review study has shown evidence that mainly severe to profound ISSHL demonstrate more hearing recovery than moderate or mild. Therefore, a selection in the patient presentation seems advisable to attain this effect. Future randomized controlled trials are advocated to further illuminate these findings before drawing any conclusions regarding HBO effectiveness as an additive treatment. Hyperbaric oxygen chambers are mostly available in specialised hospitals. Also, the costs of hyperbaric treatment modalities have to be taken into account. A facility in Australia calculated that the cost of one HBO session would be around 304 Australian Dollars [39].

### Pathophysiology ISSHL and rationale HBOT

There is a significant higher impact of the combination therapy (SS + HBO) on ISSHL patients diagnosed with severe or profound hearing loss. Various studies have shown the hearing recovery in this specific category of patients [15–21, 23–30]. In a study conducted by Topuz et al., the intervention group had a statistical significant difference in comparison to the control group in patients with initial hearing loss levels of 61–80 and ≥ 81 dB (*p* = 0.014 and *p* = 0.005,respectively) [15]. These results (amongst others) insinuate that the addition of HBO might be of benefit in cases of severe and profound hearing impairment. One might postulate that in these patients, the oxidative stress (as is mentioned before) is more elevated (and therefore damage to inner ear structures such as organ of Corti with its inner/outer hair cells), in comparison to the other groups and therefore explain the more successful outcome when HBO therapy is added to the treatment regimen. Therefore, our hypothesis finds support in that early treatment might be primordial to treat the damage (in parallel with patients after exposure to loud noise or ototoxic agents). However, no concordant evidence is available in the literature to support this.

Lamm et al., suggested HBO therapy as an adjuvant form of treatment after failure of conventional therapy in ISSHL [33]. Nonetheless, using HBO therapy as a primary treatment seems to cause less effect in comparison to the usage of steroids [27]. Various reports have indicated that the utilisation of HBO as a “last resort” remedy can cause significant improvement of hearing thresholds [33, 40–43]. The impact of spontaneous recovery should be taken into consideration while assessing the impact of HBO as a form of “salvage” therapy. According to Mattox, 29–78% of the ISSHL patients recover spontaneously in their first 2 weeks after onset [4]. A number of determinants influence the prospect of spontaneous recovery, these include degree of hearing loss, patient age, vertigo and time between onset of hearing loss and treatment [44].

### Safety and complication rate of HBOT

HBO is recognised as a safe treatment modality if the pressure is kept lower than three atmospheres (ATM) with sessions lasting up to 2 h [45]. A study conducted by Hadanny included 2334 patients with a total of 62,614 hyperbaric sessions [46]. 406 of the 2334 patients (17.4%) experienced one or more complications during their HBO sessions. with an overall per-session incidence of 721:100,000 events (0.72%). The complications did not cause any harm on a longer timeframe. Most patients endured barotrauma as a complication after initiation of the therapy. Strict protocols, such as in-chamber monitoring have to be established to ensure the safety of patients [46].

### Limitations

HBOT was given to patients with ISSHL next to steroid medication (either systemic, local or a combination of these). The variation in steroid regimen, method of administration and therapy duration differed distinctively among the studies. Also, the patients were not pooled correctly in some cases. These distinctions could potentially influence the comparability of various study cases and cause confounding bias. Though little is known about the final treatment outcome between the various application methods, it is highly unlikely the application method will interfere with hearing outcome [47]. Most of the included studies did not attest as qualitative articles. The applied guidelines for the distinction between profound and moderate ISSHL, demonstrated a notable difference. Whereas, the reports of audiometric outcomes showed some discrepancy with regards to the meaning of a 20 dB recovery for moderate ISSHL and severe to profound ISSHL. The limitations mentioned in this systematic review should be considered when evaluating its outcomes.

## Conclusion

In general, there was no significant difference observed between the intervention and control group. However, in severe to profound hearing loss, according to some reports, the patients might benefit from the adjunction of HBO to corticotherapy. Future prospective research, with larger series is required to further illuminate this topic.

## Appendix 1: Search strategy

**Table:**

Database	Search—19 May 2018
Pubmed	((((((((hearing loss, sensorineural[MeSH Terms]) OR hearing loss, sudden[MeSH Terms]) OR sudden deafness[MeSH Terms]) OR hearing impairment[MeSH Terms]) OR idiopathic sudden sensorineural hearing loss[Title/Abstract]) OR sensorineural hearing loss[Title/Abstract])) AND (((((Oxygen Therapy, Hyperbaric[MeSH Terms]) OR Oxygen Therapies, Hyperbaric[MeSH Terms]) OR Hyperbaric Oxygenations[Title/Abstract]) OR HBO[Title/Abstract]) OR hyperbaric[Title/Abstract])) AND (((((steroid*[MeSH Terms]) OR glucorticoid[MeSH Terms]) OR prednison*[MeSH Terms]) OR corticosteroids[MeSH Terms]) OR dexamethasone[Title/Abstract]) OR deltasone[Title/Abstract])
Embase	sensorineural hearing loss’:ti,ab,kw OR ‘sudden hearing loss’:ti,ab,kw OR ‘sudden deafness’:ti,ab,kw OR ‘hearing impairment’:ti,ab,kw OR ‘idiopathic sudden sensorineural hearing loss’:ti,ab,kw AND hyperbarc oxygen therapy’:ti,ab,kw OR ‘hyperbaric oxygen therapies’:ti,ab,kw OR ‘hyperbaric oxygenations’:ti,ab,kw OR ‘hbo’:ti,ab,kw OR ‘hyperbaric’:ti,ab,kw AND steroid*’:ti,ab,kw OR ‘glucocorticoid*’:ti,ab,kw OR ‘prednisone’:ti,ab,kw OR ‘corticosteroid’:ti,ab,kw OR ‘dexamethasone’:ti,ab,kw
Cochrane	“sensorineural hearing loss”:ti,ab,kw or “sudden hearing loss”:ti,ab,kw or “sudden deafness”:ti,ab,kw or hearing impairment:ti,ab,kw or idiopathic sudden sensorineural hearing loss:ti,ab,kw AND hyperbarc oxygen therapy:ti,ab,kw or “hyperbaric oxygen therapies”:ti,ab,kw or “hyperbaric oxygenation”:ti,ab,kw or “HBO”:ti,ab,kw or “hyperbaric”:ti,ab,kw AND “steroid”:ti,ab,kw or “glucocorticoid”:ti,ab,kw or “prednisone”:ti,ab,kw or “corticosteroid”:ti,ab,kw or “dexamethasone”:ti,ab,kw
